# *Hydraena* Kugelann, 1794 (Coleoptera, Hydraenidae) from the Seychelles, Indian Ocean, with description of a new species

**DOI:** 10.3897/zookeys.623.10052

**Published:** 2016-10-11

**Authors:** Manfred A. Jäch, Juan A. Delgado

**Affiliations:** 1 Naturhistorisches Museum Wien, Burgring 7, A – 1010 Wien, Austria; 2 Departamento de Zoología, Facultad de Biología, Universidad de Murcia, E – 30100 Murcia, Spain

**Keywords:** Coleoptera, Hydraenidae, Hydraena, new species, Seychelles, Mahé, La Digue, Silhouette, Indian Ocean

## Abstract

*Hydraena matyoti* **sp. n.** (Coleoptera, Hydraenidae) is described from the Seychelles, Indian Ocean. *Hydraena mahensis* Scott, 1913 is redescribed. The latter is here recorded from La Digue for the first time. A key to the species of the genus *Hydraena* Kugelann, 1794 of the Seychelles is presented.

## Introduction

So far, only one species of Hydraenidae, *Hydraena mahensis* Scott, 1913, has been recorded from the Seychelles (see Hansen 1998, Jäch and Madl 2009). However, when Jäch and Madl (2009) summarized the water beetle fauna of the Seychelles they were not aware that the species from Mahé, which they had photographed (Jäch and Madl 2009: Fig. 15), was actually an undescribed one. Only five years later, when Michael Madl rediscovered numerous specimens of the true *Hydraena mahensis* on Mahé Island did they realize this error. In the present paper *Hydraena mahensis* is redescribed, and the second species is described as new for science.

## Material and methods

Line drawings were prepared with the aid of a *camera lucida* attached to a Nikon eclipse E600 microscope. Habitus photographs were taken with a Nikon DS-U2 unit Camera attached to a Leica MZ9S stereomicroscope. Images were stacked using CombineZP.

### Abbreviations

BMNH
 The Natural History Museum, London, UK 


CDUM
 Coll. J.A. Delgado, University of Murcia, Spain 


IBE
 Institute of Evolutionary Biology (Institut de Biologia Evolutiva), Barcelona, Spain 


NMW
 Naturhistorisches Museum Wien, Austria 


## Taxonomy

### Hydraena (Hydraenopsis) mahensis

Taxon classificationAnimaliaColeopteraHydraenidae

Scott, 1913

Figs 1 , 3–6 , 7–11

Hydraena (s.str.) mahensis : Scott 1913: 196; Knisch 1924: 39; Hansen 1998: 49.
Hydraena mahensis : Marlier 1979: 53.
Hydraena (Hydraenopsis) mahensis : Jäch et al. 2000: 80; Jäch and Madl 2009: 19 (*partim*).

#### Type localities.

Marshes of coastal plain at Anse aux Pins and Anse Royale, Mahé, Seychelles.

#### Type material.

Two syntypes. One of these syntypes is deposited in the BMNH (M. Barclay, email, 22.VII.2016). It is labelled as “Holotype” (red edged disc) and as ‘Type’ (blue rectangle). The second syntype (“Mahe 146” [handwritten on the rear edge of the card carrying the beetle], “Mahe 1908-09 Seychelles Exp.” [printed], “Hydraena mahensis H. Scott Paratype.” [handwritten]) is deposited in the Cambridge University Museum (W. Foster, email, 7.IX.2016).

#### Material examined.

Mahé: 26 exs. (CDUM, NMW): Mahé (south), Petite Police Bay, swamp, 4°48.10'S 55°31.03'E, IX.2014, leg. M. Madl. La Digue: 1 ♂, 1 ♀ (NMW): La Réunion, ca. 5 m a.s.l., 7.IV.2007, leg. G. Wewalka (7).

The DNA of one female (voucher number IBE-AN186) was non-destructively extracted using the DNeasy Tissue Kit (Qiagen GmbH, Hilden, Germany) in the IBE. Two fragments of the cytochrome C oxydase subunit (COI) were sequenced, the 5’ end (the barcode fragment, primers LCO1490 and HCO2198, Folmer et al. 1994) and the 3’ end (primers Jerry-M202 and Pat-M70, Simon et al. 1994), and submitted to GenBank with accession numbers LT593860 and LT593861 respectively. The extracted specimen and DNA are deposited in the IBE.

#### Redescription.

**Figure 1.**
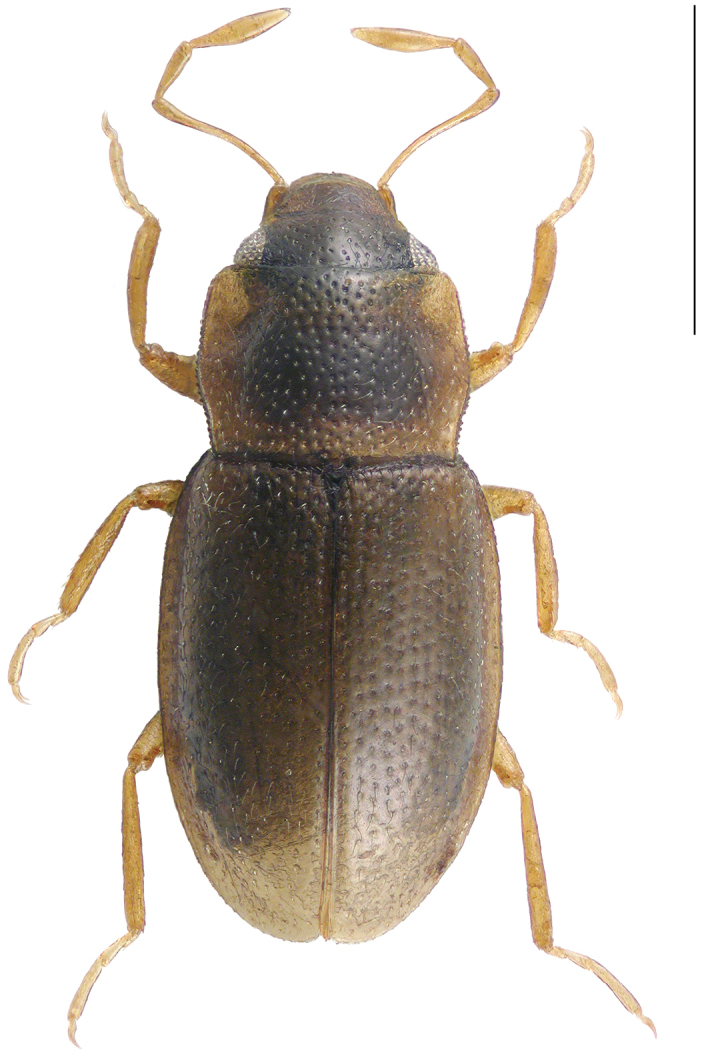
Habitus of *Hydraena mahensis* Scott, 1913, male. Scale bar: 0.5 mm.

Habitus as in Fig. 1. Body length (without abdomen): 1.20–1.40 mm. Dorsum brown, frons dark brown to black, posterior and lateral sides of pronotum paler yellowish brown; maxillary palpi and legs yellowish to yellowish brown.

Labrum deeply excised anteriorly; lobes rounded anteriorly. Middle of clypeus very sparsely punctate and glabrous, lateral parts densely micropunctate and mat. Fronto-clypeal suture more or less straight, slightly impressed. Frons moderately densely punctate laterally, sparsely punctate medially, interstices shining; interocular grooves obsolete. Eyes large, protruding, more than 30 facets visible in dorsal view.

Pronotum wider than long, widest near middle; anterior margin weakly concave; anterior angles rounded; lateral rim denticulate; surface moderately densely punctate, but disc sometimes more sparsely punctate; discal foveae more or less obsolete.

Elytra elongately oval; with about nine rows of punctures between suture and shoulder; punctures small, not deeply impressed, arranged in almost regular, usually not impressed lines; intervals and interstices flat and glabrous; explanate margin of elytra only moderately wide, not strongly serrate posteriorly. Elytral apices usually separately rounded.

Foretibia very slightly curved in both sexes.

Mesoventral process parallel-sided, apically truncate, width sexually dimorphic. Metaventrite moderately deeply impressed in the middle; metaventral plaques rather indistinct, sometimes obscured by dense punctures.

Male terminal sternite and spiculum (Fig. 7): Sternite firmly connected with spiculum, subrectangular, almost twice as long as wide, almost symmetrical, with small subapical cavity; base with very small lateral projections.

Aedeagus (Figs 3–6): Total length: 190 µm. Main piece short, almost straight, with two moderately long setae, and a few very short ones on left side near base of distal lobe; phallobase slightly asymmetrical, closed proximally. Distal lobe quite large, about as long as main piece, forming a distinct angle with main piece (in lateral view), apically furcate. Right paramere wide, elongate, about half as long as main piece, articulately connected with main piece, inserted near apex of main piece; with four long apical setae and four moderately long setae on ventral face along right margin. Left paramere similar to right one, but slightly shorter, firmly connected with main piece, inserted on left side of apex.

Gonocoxite (Fig. 8): Subpentagonal, transverse; lateral margins curved; basal apophyses short; inner plate not projecting.

Female tergite X (Fig. 9): Subtrapezoidal, transverse; disc sparsely covered with trichoid setae; subapical setae vermiform; apical margin excised medially.

Spermatheca (Figs 10–11): Proximal portion crescentic; distal portion cup-shaped; ductus apically wrinkled.

Secondary sexual characters: Male mesoventral process more slender; in male more or less as wide as mesotibia, in female slightly wider than mesotibia.

#### Habitat.

Swamps and ditches in coastal plain. The swamp at Petite Police Bay (Fig. 20) dries out periodically.

#### Distribution.

This species is so far known only from Mahé (Anse aux Pins, Anse Royale, Petite Police Bay) and La Digue (La Réunion). It is here recorded from La Digue for the first time.

### Hydraena (Hydraenopsis) matyoti

Taxon classificationAnimaliaColeopteraHydraenidae

Jäch & Delgado sp. n.

http://zoobank.org/510E6CEA-96B4-484F-939C-ED99D955C58E

Figs 2 , 12–14 , 15–19

Hydraena (Hydraenopsis) mahensis : Jäch & Madl 2009: 19 (*partim*), 29.

#### Type locality.

Small puddle on Sans Souci Hiking Trail, northern Mahé, Seychelles.

#### Type material.

**Holotype** ♂ (NMW), glued on pinned card, genitalia extracted and glued on same card. Label data: “SEYCHELLES: Mahé Sans Souci, XI. 1994, leg.E.Heiss”. **Paratypes**: 1 ♀ (NMW): same label data as holotype; 1 ♂ (NMW): “SEYCHELLES: Mahé Morne Seychellois NP Casse Dent, trail 25.03.2011 leg. M.Madl”; 2 ♀♀ (NMW): “SEYCHELLEN, Mahé 1996 Morne Seychellois NP Congo Rouge 600-800m 25. V. leg. Schödl (12c)”; 1 ♀ (NMW): “Seychelles, Silhouette, Jardin Marron [field name], 400m, 12. 4. 2007, leg. Wewalka (12)”.

#### Description.

**Figure 2.**
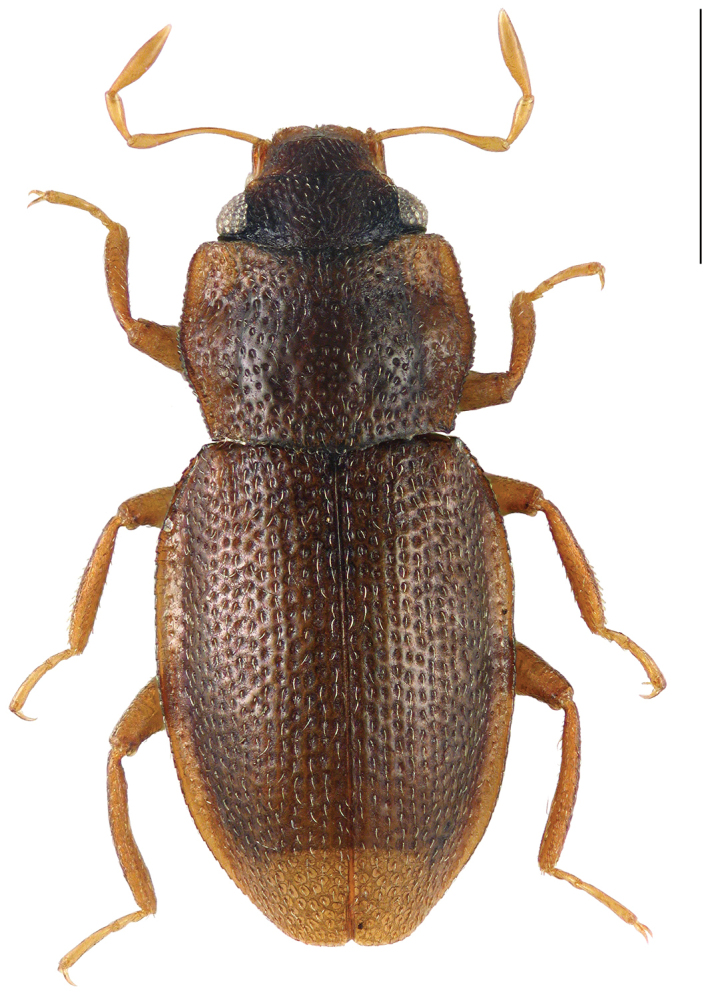
Habitus of *Hydraena matyoti* Jäch & Delgado, sp. n., holotype, male. Scale bar: 0.5 mm.

Habitus as in Fig. 2. Body length (without abdomen): 1.56–1.70 mm. Dorsum reddish brown, lateral parts of frons (near eyes) more or less black; maxillary palpi yellowish, apical tip paled.

Labrum deeply excised anteriorly; anterior angles rounded. Middle of clypeus sparsely punctate, usually glabrous, lateral parts usually micropunctate and mat. Fronto-clypeal suture straight or feebly arcuate, slightly impressed. Frons moderately densely (middle) or more densely and sometimes even rugosely (laterally) punctate, interstices shining; interocular grooves shallow. Eyes large, protruding, more than 30 facets visible in dorsal view.

Pronotum distinctly wider than long, widest near middle; anterior margin concave; anterior angles rounded; lateral margin very slightly concave in anterior and posterior half; lateral rim denticulate; surface moderately densely to densely punctate, but disc sometimes only sparsely punctate; anterior discal foveae obsolete, posterior discal foveae hardly noticeable.

Elytra elongately oval; with about nine rows of punctures between suture and shoulder; punctures small, but rather deeply impressed, usually arranged in regular, usually not impressed lines; intervals sometimes convex, glabrous; explanate margin of elytra comparably wide, abruptly attenuate subapically, slightly to distinctly serrate posteriorly. Elytral apices usually separately rounded.

Foretibia and metatibia sexually dimorphic.

Mesoventral process parallel-sided, apically truncate, width sexually dimorphic. Metaventrite moderately deeply impressed between glabrous metaventral plaques, the latter reduced to very thin glabrous elevated short, widely separated streaks.

Male terminal sternite and spiculum (Fig. 15): Sternite not firmly connected with spiculum, subtrapezoidal, approximately as wide as long, slightly asymmetrical; base with very small lateral projections.

Aedeagus (Figs 12–14): Total length: 400 µm. Main piece elongate, in apical half divided into a ventral and a dorsal branch, ventral branch with characteristic claw-like apex; single dorsal seta inserted on dorsal branch near base of distal lobe; phallobase strongly asymmetrical, closed proximally. Distal lobe inserted on dorsal branch of main piece; moderately large, amorphic, partly distinctly hyaline. Right paramere long and slender, with rows of subapical setae; articulately connected with main piece. Left paramere absent.The aedeagus can be distinguished from the aedeagi of *Hydraena borbonica* Fairmaire, 1898 (from La Réunion) and *Hydraena ofella* Balfour-Browne, 1958 (from the Comoros) by the wider and less regular shape of the ventral branch of the main piece.

Gonocoxite (Fig. 16): Subtrapezoidal, strongly transverse; basal part without setae, distal part strongly setose; basal apophyses small; inner plate slightly projecting.

Female tergite X (Fig. 17): Subsemicircular, transverse; disc sparsely covered with trichoid setae; subapical setae vermiform; apical margin without excision.

Spermatheca (Figs 18–19): Proximal portion crescentic; distal portion elongately cup-shaped.

Secondary sexual characters: Foretibia and metatibia slightly curved in male. Male mesoventral process more slender; in male more or less as wide as mesotibia, in female slightly wider than mesotibia.

**Figures 3–6.**
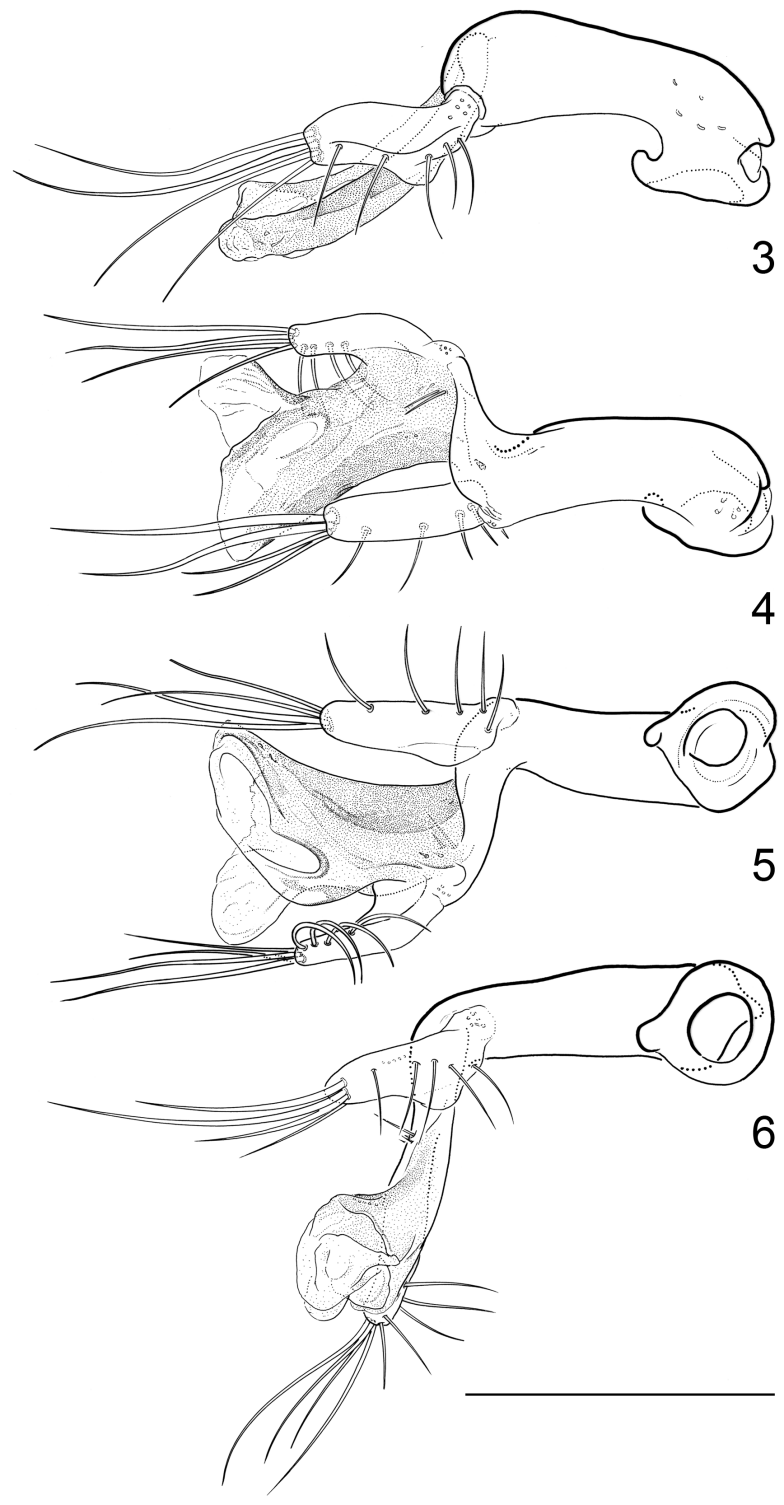
*Hydraena mahensis* Scott, 1913, aedeagus: **3** strictly lateral view **4** dorsal view **5** strictly ventral view **6** ventral view, slightly rotated to left side. Scale bar: 0.1 mm.

**Figures 7–11.**
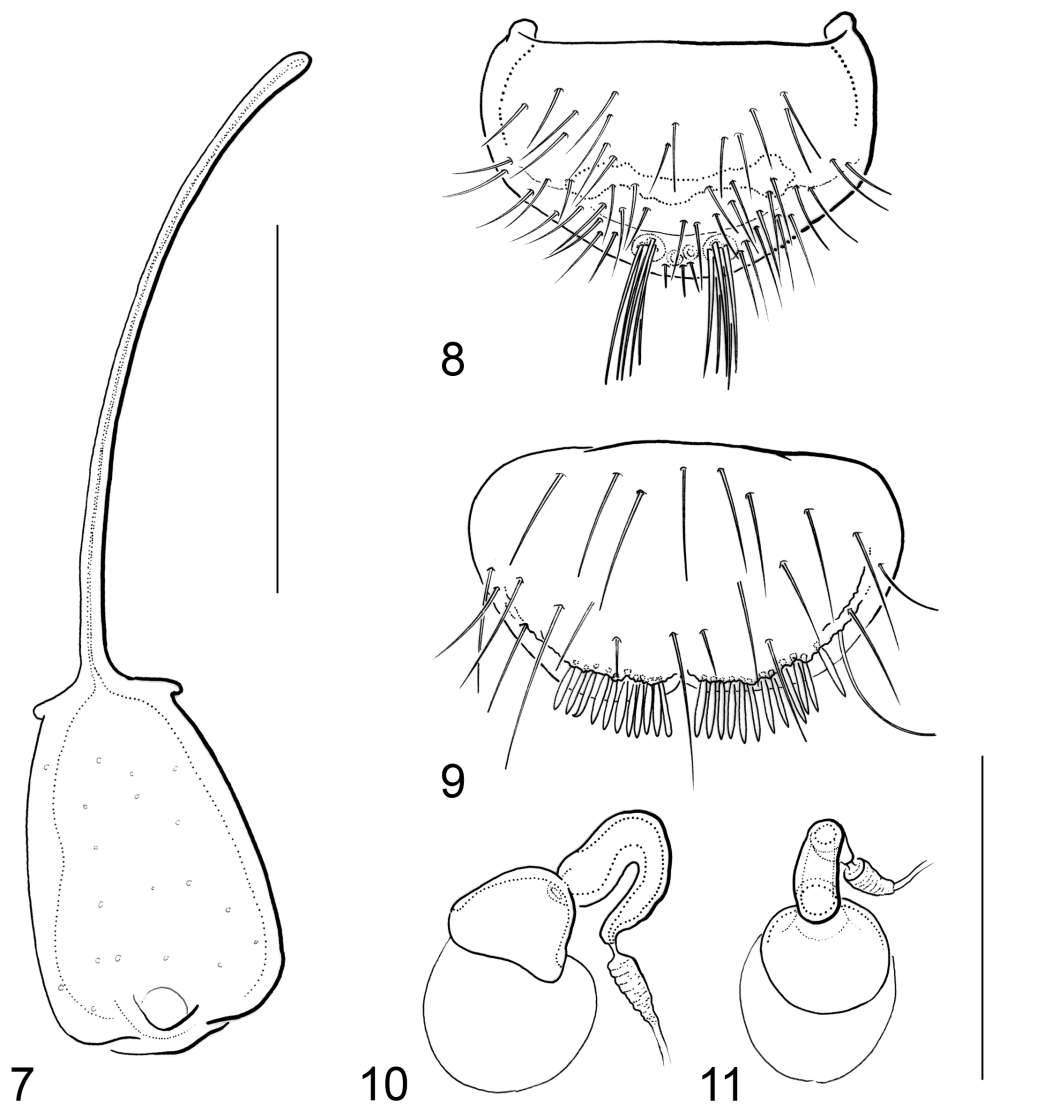
*Hydraena mahensis* Scott, 1913: **7** male terminal sternite and spiculum **8** gonocoxite **9** female tergite X **10–11** spermatheca, in different views. Scale bar: 0.1 mm.

**Figures 12–14.**
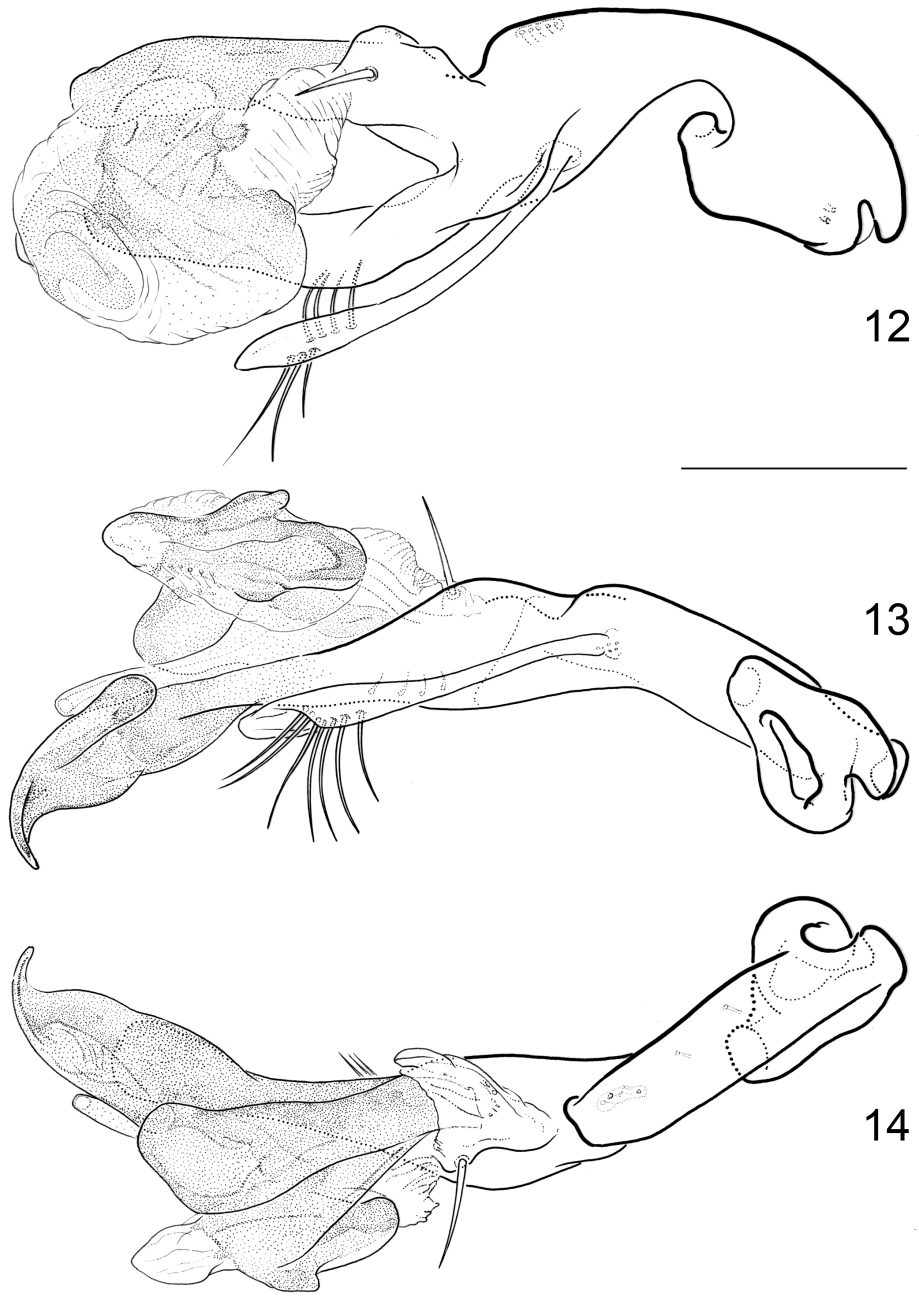
*Hydraena matyoti* Jäch & Delgado, sp. n., aedeagus: **12** lateral view **13** ventral view **14** dorsal view. Scale bar: 0.1 mm.

**Figures 15–19.**
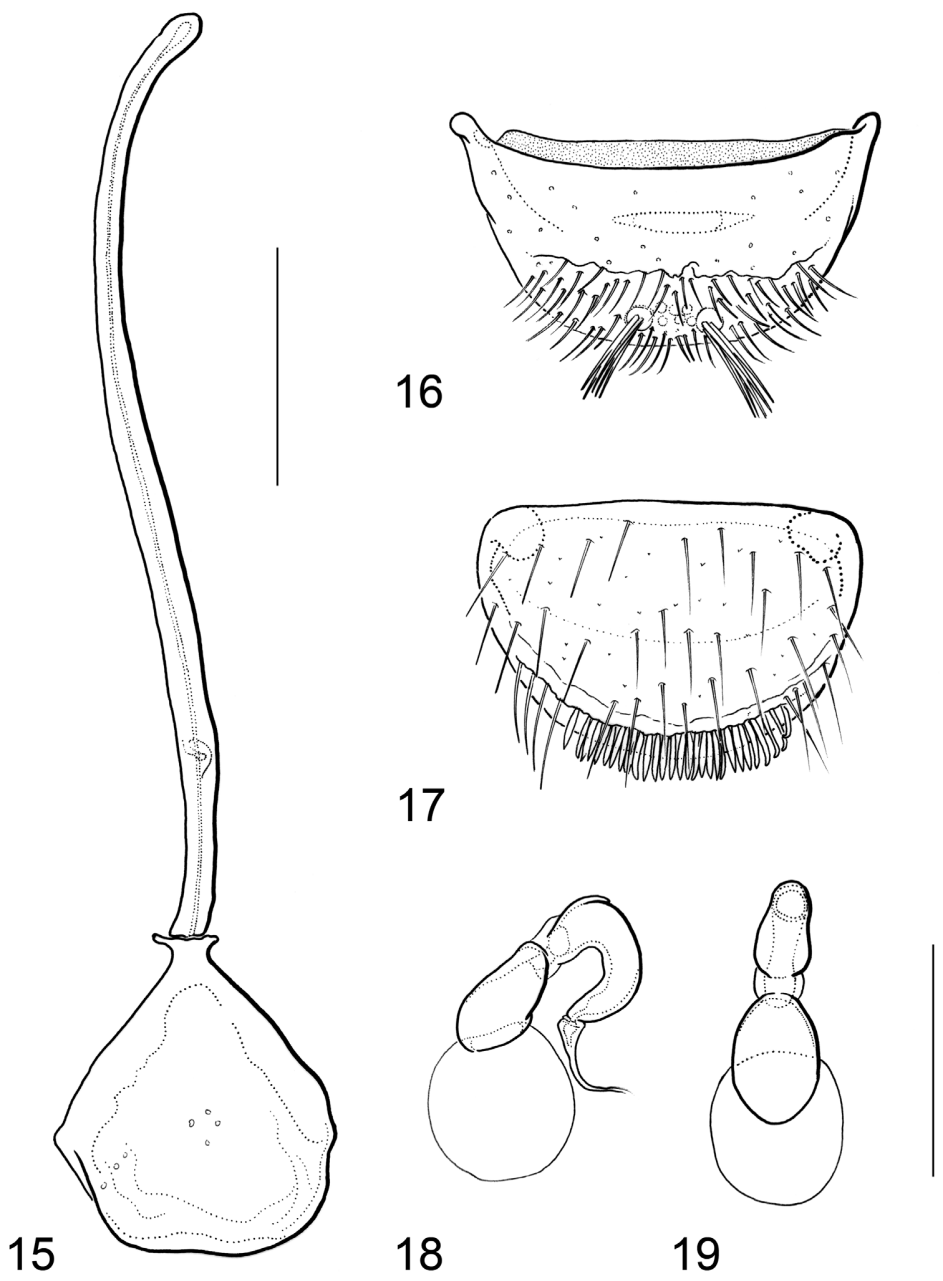
*Hydraena matyoti* Jäch & Delgado, sp. n.: **15** male terminal sternite and spiculum **16** gonocoxite **17** female tergite X **18–19** spermatheca, in different views. Scale bar: 0.1 mm.

#### Habitat.

On Mahé this species was collected in a small puddle on a forest trail (leg. E. Heiss), and in small mountain streams at more than 600 m a.s.l. (leg. M. Madl and S. Schödl) – the single specimen collected by M. Madl was found on a small piece of wood lying in a very small stream (Fig. 21). A single female was collected on Silhouette, Jardin Marron, near a hiking trail, ca. 400 m a.s.l., approx. 4°29.16'S 55°14.16'E, on a hygropetric rockface (leg. G. Wewalka).

**Figure 20.**
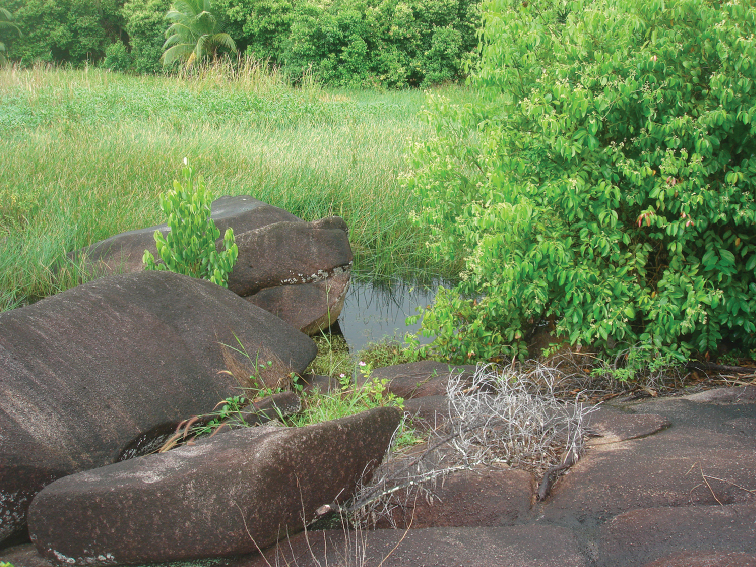
Habitat of *Hydraena mahensis* Scott, 1913. Swamp at Petite Police Bay, Mahé.

**Figure 21.**
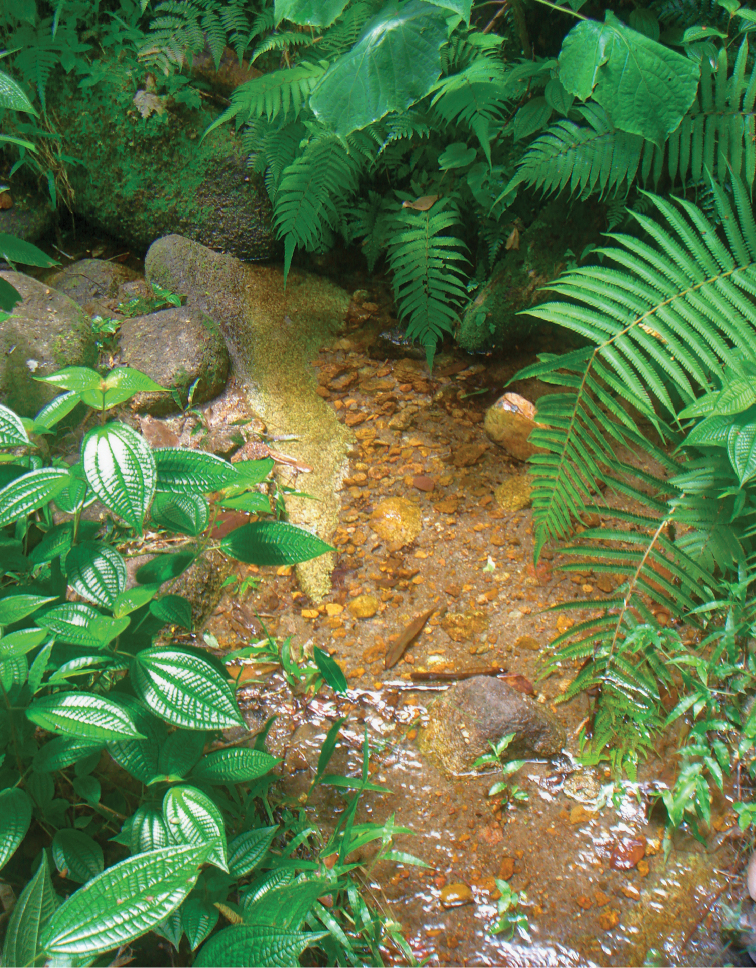
Habitat of *Hydraena matyoti* Jäch & Delgado, sp. n. Small stream, Morne Seychellois National Park, Mahé.

#### Distribution.

Endemic to the Inner Seychelles. So far known only from Mahé and Silhouette.

#### Etymology.

This species is named for Pat Matyot, a Seychellois naturalist with a special interest in entomology. Pat Matyot is employed by the Seychelles Broadcasting Corporation and has produced many television features on the country’s fauna and flora. He has served on the boards and science committees of a number of conservation organisations in Seychelles and is at present a board member of the Island Conservation Society (ICS) and the Silhouette Foundation. The epithet is a noun in the genitive case.

### Key to the species of *Hydraena* of the Seychelles

**Table:** 

1	Body length (without abdomen): 1.20–1.40 mm. Frons without interocular depressions (Fig. 1). Foretibia of female slightly curved. Metatibia of male straight (Fig. 1). Explanate margin of elytra narrow (Fig. 1). Male terminal sternite firmly connected with spiculum, subrectangular (Fig. 7). Aedeagus (Figs 3–6) very small (190 µm long), more or less y-shaped in ventral/dorsal view, with two parameres. Female tergite X (Fig. 9) excised apically	***mahensis***
–	Body length (without abdomen): 1.56–1.70 mm. Frons with shallow interocular depressions (Fig. 2). Foretibia of female straight. Metatibia of male slightly curved (Fig. 2). Explanate margin of elytra comparably wide, abruptly attenuate subapically (Fig. 2). Male terminal sternite not firmly connected with spiculum, subtrapezoidal (Fig. 15). Aedeagus (Figs 12–14) distinctly larger (400 µm long), not y-shaped in ventral/dorsal view, with one slender paramere. Female tergite X (Fig. 17) not excised apically	***matyoti***

## Discussion

The two *Hydraena* species of the Seychelles obviously live in different habitats. While *Hydraena mahensis* is known only from lowland stagnant water, i.e., coastal swamps near the sea, the new species, *Hydraena matyoti*, was collected only at higher elevations in the mountainous interior of the Seychelles Islands, i.e., in a small puddle, mountain streams, and in a seepage on a cliff. In suitable habitats, *Hydraena mahensis* can be found in abundance, while *Hydraena matyoti* seems to be generally very rare. In total, only six specimens were collected between 1994 and 2011 by four Austrian entomologists.

Although both species belong to the same subgenus they are not closely related and in fact they represent different species groups. *Hydraena mahensis* is very closely related to *Hydraena erythraea* Régimbart, 1905 (*Hydraena erythraea* group; “*erythræa*-phylum” sensu Balfour-Browne 1950: 11), described from Eritrea. The aedeagi of these two species are characterized by the very small size, the angulate form, as well as the position and shape of the parameres. The right paramere of *Hydraena erythraea* is distinctly smaller than in *Hydraena mahensis*. The *Hydraena erythraea* group is wide-spread in East Africa. *Hydraena matyoti* is probably related to *Hydraena borbonica* and *Hydraena ofella* (*Hydraena borbonica* group). The aedeagi of these three species possess a deeply furcate main piece with a single dorsal seta inserted on dorsal branch near base of distal lobe, and with a single elongate and slender paramere on the right side. Possibly, *Hydraena balfourbrownei* Bameul, 1986 and *Hydraena legorskyi* Jäch & Brojer, 2012 also belong to this group. Although the aedeagi of these two species possess a very long and slender left paramere, the deeply furcate main piece and the shape and position of the right paramere suggest a close relationship.

## Supplementary Material

XML Treatment for Hydraena (Hydraenopsis) mahensis

XML Treatment for Hydraena (Hydraenopsis) matyoti
